# Cavitation in blunt impact traumatic brain injury

**DOI:** 10.1007/s00348-024-03853-6

**Published:** 2024-07-17

**Authors:** John D. Finan, Thea E. Vogt, Yasaman Samei

**Affiliations:** Department of Mechanical and Industrial Engineering, University of Illinois Chicago, Chicago, IL USA

## Abstract

Traumatic brain injury (TBI) poses a major public health challenge. No proven therapies for the condition exist so protective equipment that prevents or mitigates these injuries plays a critical role in minimizing the societal burden of this condition. Our ability to optimize protective equipment depends on our capacity to relate the mechanics of head impact events to morbidity and mortality. This capacity, in turn, depends on correctly identifying the mechanisms of injury. For several decades, a controversial theory of TBI biomechanics has attributed important classes of injury to cavitation inside the cranial vault during blunt impact. This theory explains counter-intuitive clinical observations, including the coup–contre-coup pattern of injury. However, it is also difficult to validate experimentally in living subjects. Also, blunt impact TBI is a broad term that covers a range of different head impact events, some of which may be better described by cavitation theory than others. This review surveys what has been learned about cavitation through mathematical modeling, physical modeling, and experimentation with living tissues and places it in context with competing theories of blunt injury biomechanics and recent research activity in the field in an attempt to understand what the theory has to offer the next generation of innovators in TBI biomechanics.

## Introduction

Traumatic brain injury (TBI) remains a common, devastating, and incurable condition that is only growing in significance in the US and worldwide. In the US, TBI distributes across ages in a trimodal fashion, with incidence peaking among young children, teenagers, and the elderly (Cancelliere et al. 2017). The number of TBIs among the elderly is increasing as the population ages (Giner et al. 2022), which is particularly concerning because older people have a poorer prognosis (Yang et al. 2022). Simultaneously, the incidence of TBI due to road traffic accidents is increasing worldwide as more people gain access to automobiles (Jiang et al. 2023). Most TBIs are mild (these injuries are also known as concussions). While the acute symptoms of mild TBI typically resolve quickly and completely, a substantial minority of patients experience a collection of sustained symptoms known as post-concussion syndrome (Cancelliere et al. 2023). What is more, mild TBI increases the risk of neurodegenerative conditions including Alzheimer’s disease (Barnes et al. 2014) and Parkinson’s disease (Gardner et al. 2015). Even head impacts that cause no acute symptoms (also known as sub-concussive impacts) may cause cumulative damage that increases the risk of neurodegeneration (McKee et al. 2009). No therapies exist for TBI sequelae so technologies that prevent or minimize damage at the time of trauma play a critical role. These technologies include helmets, airbags, and various other protective devices. Our incomplete understanding of the mechanical tolerance of the human brain limits our ability to optimize these devices. The key to understanding tolerance is understanding the mechanisms of injury. These mechanisms may vary across the different classes of TBI, which are blunt impact TBI, penetrating TBI, and blast TBI. Penetrating TBI and blast TBI pose important, unsolved challenges. However, this review will focus on blunt impact TBI, and the role of a controversial injury mechanism called cavitation. Most of the early work on cavitation as an injury mechanism applied it to blunt impact TBI but, in recent years, cavitation has more frequently been applied to blast TBI. The goal of this review is to summarize what is now known about cavitation in TBI and re-evaluate the role of cavitation in blunt impact TBI in light of this knowledge.

## The development of the cavitation theory of TBI

Cavitation was well understood long before it was applied to TBI. Water vaporizes when the temperature and/or pressure changes. Boiling is vaporization of water at constant pressure due to increasing temperature. The alternative scenario—vaporization at constant temperature due to decreasing pressure—is called cavitation (Brennen 1994; Plesset & Calif 1949; Rayleigh 1917). As pressure drops, bubbles spontaneously nucleate, grow, and collapse. As a bubble collapses, small perturbations of the ideal spherical shape grow into fast-moving microjets of liquid. Shock waves also emanate from the collapsing bubble. The combined action of these microjets and shock waves can damage even strong, metallic surfaces in the vicinity (Dular et al. 2019). It also generates noise. The US Navy sponsored important early work on cavitation because it can occur on the trailing faces of propellors, damaging them (Plesset & Ellis 1955) and making them audible to the enemy (Mellen 1954). While cavitation noise may be problematic in combat, it is useful in the laboratory because it allows hydrophones to detect cavitation (Eckersley et al. 2020, 2021). In all circumstances, a decline in pressure relative to ambient conditions causes cavitation. Negative relative pressures in the cranial vault during head impacts provided the first clue that cavitation might play a role in TBI.

**Fig. 1:**
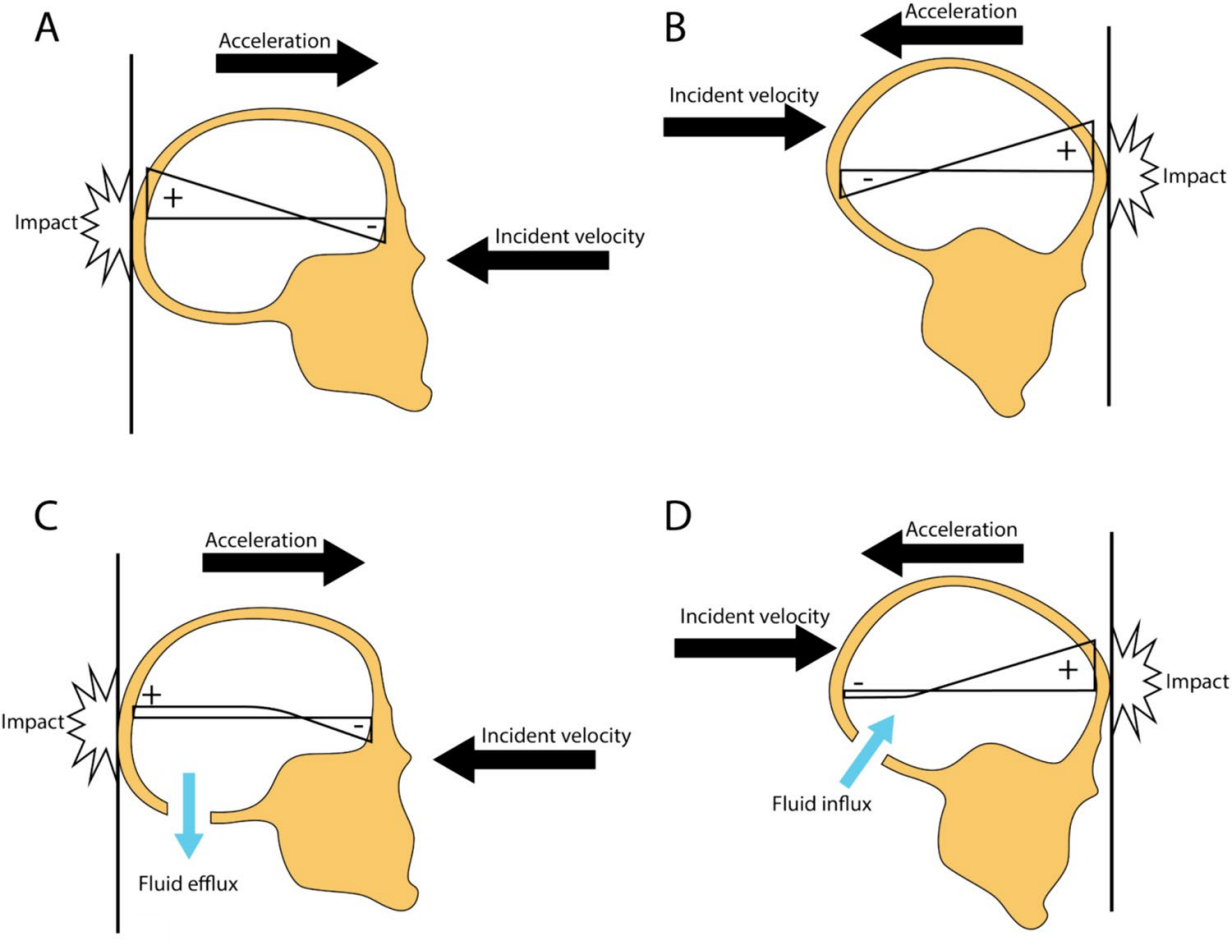
Pressure distributions in the cranial vault during impact. **A** If the cranial vault is modeled as a closed vessel, cavitation theory predicts a positive pressure pulse underneath the point of impact in an occipital collision and a declining pressure distribution along the line of impact force that culminates in a negative pressure pulse underneath the frontal surface of the cranial vault. Cavitation theory predicts contusions will form at this location, which is consistent with autopsy data. **B** If the cranial vault is modeled as a closed vessel, a frontal collision creates a negative pressure pulse underneath the occipital surface of the cranial vault. Cavitation theory predicts contusions will form at this location, which is not consistent with autopsy data. Critics of cavitation theory consider this inconsistency an important failure. **C** Proponents of cavitation theory point out that the cranial vault is not in fact closed. A small amount of fluid flow can occur through the foramen magnum and this flow diminishes positive and negative pressure pulses in the occipital part of the cranial vault. During an occipital impact, fluid efflux through the foramen magnum diminishes the positive occipital pressure pulse. However, the negative pressure pulse in the frontal part of the cranial vault, far from the foramen magnum, persists and causes contusions via cavitation. **D** In a frontal impact, fluid rushes in through the foramen magnum as the pressure falls in the occipital part of the cranial vault, preventing pressure from falling low enough to cause cavitation and explaining why contusions are not observed here

Early work with cadavers and animals used strain gages and pressure sensors to demonstrate that head impact bends the skull and generates transient positive and negative relative pressures in the cranial vault (Goggio 1941; Gurdjian & Lissner 1944; Gurdjian et al. 1953; Gurdjian & Webster 1947; Thomas et al. 1967). In 1948, Ward hypothesized that impact-induced negative pressures led to cavitation, which, in turn, injured cells (Ward et al. 1948). Gross expanded this concept in 1958 to explain the coup–contre-coup pattern of injury commonly observed in TBI patients (Gross 1958a). In a coup–contre-coup injury, a contusion or bruise occurs in the cortex near the impact (the coup site), and another contusion occurs on the opposite side of the brain (the contre-coup site). Gross demonstrated that striking one end of a sealed vessel full of water induced cavitation at the opposite end and hypothesized that the skull and brain behave like the glass vessel and contained fluid, respectively, during an impact. His model attributed contusions to the spatial and temporal variation of pressure in the cranial vault as follows: The skull moves away from the impact, creating tension that leads to negative pressure in the cerebrospinal fluid (CSF) and brain tissue at the contre-coup site. When the impact ends, the skull rebounds elastically at the point of impact, creating tension that leads to negative pressure at the coup site (see Fig. 1A). Gross predicted that these negative pressure pulses would cause cavitation that locally injured the cortex, creating contusions. Perfectly pure water has cohesive strength so substantial negative absolute pressure is required to cause cavitation. However, small concentrations of impurities can nucleate cavitation bubbles and drop the negative absolute pressure threshold for cavitation to almost zero. Gross assumed that fluids in the brain contained such impurities so cavitation occurs as soon as pressure dips below absolute zero.

He also predicted that TBI is more likely at high altitude because cavitation requires less negative relative pressure when the ambient atmospheric pressure is lower (Gross 1958b). A comparison of high school football players in Colorado and Florida found increased incidence of TBI at high altitude (Li et al. 2022) as Gross predicted. While this finding aligns with Gross’s prediction, it remains unclear if cavitation is indeed the underlying mechanism. High altitude causes a number of changes in the brain, including brain swelling, that could influence traumatic injury brain biomechanics (Shushanyan et al. 2024).

## Analytical models of cavitation

Local pulses of negative relative pressure trigger cavitation, so validation of the cavitation theory requires an understanding of how pressure varies over space and time in the cranial vault during an impact. The first analytical models represented the skull as a rigid shell and the brain as a Newtonian fluid (Anzelius 1943). These models predicted that pressure would peak at the center of the skull. However, the assumption of a rigid skull was a critical flaw (Goldsmith 1966) and subsequent models instead represented the skull as an elastic material (Engin 1969; Hickling & Wenner 1973). This change transformed the pressure distribution, creating peaks of positive and negative pressure at the coup and contre-coup sites that agreed with the cavitation model of TBI and experimental measurements. Contre-coup contusions are counter-intuitive because they occur far from the point of impact. The capacity of cavitation theory to explain these injuries is, therefore, among its greatest attractions. Paradoxically, the spatial distribution of injury predicted by cavitation theory has also motivated skepticism (Ommaya et al. 1971).

Some critics dismissed the cavitation theory because it predicted occipital contre-coup contusions in frontal impacts (Ommaya et al. 1971). In reality, frontal impacts rarely cause contre-coup contusions (Courville 1942; Goggio 1941) (see Fig. 1). The primary alternative to cavitation theory is a model first presented by Holbourn (Holbourn 1943) in which rotational acceleration of the skull induces shear strain in the brain tissue. In this model, lesions occur predominantly in the frontal region regardless of the point of impact because the sphenoid ridge in the base of the skull projects into the brain at this location, amplifying local strains. Therefore, the shear strain model succeeded in predicting the spatial distribution of lesions because it had a biofidelic shape (the details of the skull base are particularly important (Ivarsson et al. 2002)). More recently, influential work on reconstructing head impacts in National Football League games found that head acceleration caused a peak in brain tissue deformation near the impact site and then a later peak on the opposite side of the skull, i.e., the combination of acceleration and shear strain can induce a coup–contre-coup injury pattern without cavitation (Viano et al. 2005). However, the cavitation model that failed to predict the lesion distribution was a simple analytical model that represented the head as a sphere, which is a gross approximation of its true shape (Ommaya et al. 1971). In that sense, the comparison of the two models was not made on a level playing field. In fact, Gross predicted that the head would respond differently to impacts from different directions precisely because it is not a simple symmetrical shape. To be more specific, he predicted that occipital impacts would create contre-coup lesions in the frontal region, but frontal impacts would not create contre-coup lesions in the occipital region because the foramen magnum, an opening at the back of the skull, would allow inflow of CSF (see Fig. 1C, D) that diminished negative pressure pulses (Gross 1958a). An unbiased comparison of the two theories requires more sophisticated models that can describe the influence of skull geometry on both cavitation and shear strain. During the 1970s, computational models largely replaced analytical models, allowing more accurate geometries.

## Computational models of cavitation

Head injury biomechanics involves nonlinear materials, complex geometries, and uncertain constitutive properties (Chatelin et al. 2010; Finan et al. 2012, 2017). Even with the most powerful modern tools, computational modeling of head impacts requires approximation and estimation (Finan et al. 2014). A finite element model (FEM) that modeled the skull as an ellipsoid concluded that shear strain and cavitation played roughly equal roles in impacts along the axis of symmetry, but shear strains dominated in glancing impacts (Chan 1974). These authors also argued that negative relative pressures might rupture capillaries even if they were not negative enough to induce cavitation. A modal analysis of the human skull found two modes that drove shear strains and one that induced cavitation. Activation of the cavitation-related mode depended on the duration of the impact but not its location (Nickell & Marcal 1974), which contradicts the criticism that cavitation models predict different results for different impact locations. The first 3D FEM of head injury predicted negative pressures sufficient to induce cavitation in the contre-coup region during occipital impacts. It also agreed with Gross’ prediction that the foramen magnum would suppress contre-coup negative pressures in a frontal impact (Shugar 1977). Two FEM studies that used 2D sagittal head geometries concluded that shear strain theory explained lesion location better than cavitation theory (Chu et al. 1994; Huang et al. 2000). However, both of these studies loaded the head with rotational acceleration rather than a point load so they did not induce skull flexure, which plays an essential role in cavitation mechanics (Goldsmith 1966). Park et al. applied biphasic theory to the problem (Park & Yoon 1997). Biphasic theory describes tissues such as articular cartilage in which a porous solid matrix is saturated by a fluid that can flow through it and exchange momentum with it (Mow et al. 1980). The biphasic model predicted more severe negative contre-coup pressures than a conventional monophasic model, especially in the frontal region, i.e., it agreed with cavitation theory. A 3D model that was validated against pressure measurements from cadaveric head impacts (Nahum et al. 1977) predicted that contre-coup lesions were more likely in occipital impacts than in frontal impacts, in agreement with clinical findings (Ruan et al. 1994). In summary, computational models that applied point loads and included skull flexure generally predicted negative relative pressures sufficient to induce cavitation in some impact scenarios. Also, these models frequently predicted significant shear strains and cavitation in the same impact, demonstrating that shear strains and negative pressures are not mutually exclusive. While computational models can predict negative relative pressures likely to cause cavitation, it is difficult for them to explicitly describe cavitation events because the associated physics are highly localized and nonlinear. Therefore, physical models play an essential role in observing what happens once cavitation bubbles form in head impact-like scenarios.

## Physical models of cavitation

A physical model for the study of cavitation in head impact typically involves a transparent vessel (representing the skull) filled with fluid and/or gel (representing the contents of the cranial vault). The first physical models were instrumented with pressure sensors. Negative relative pressures consistent with cavitation were recorded in a water-filled cylinder when it was accelerated (Kopecky & Ripperger 1969). In spherical shells filled with water, impact caused negative relative pressures (Kenner & Goldsmith 1973) but ultrasonic loading did not (Suh et al. 1972), again indicating that skull flexure plays a critical role. Impact also induced negative pressures at the contre-coup site in a spherical shell filled with gelatin (Edberg et al. 1963). Goldsmith introduced a surrogate neck to support the model head (Goldsmith et al. 1978; Landkof et al. 1976) and used high-speed cameras to record cavitation bubbles forming and collapsing in a scenario resembling a head impact for the first time (Lubock & Goldsmith 1980). Cavitation occurred at the interface between gelatin representing the brain and water representing the CSF, suggesting cavitation happens on the brain surface but not inside the brain. A similar study using a more biofidelic geometry also recorded cavitation on the brain surface (Lang et al. 2021). These studies assumed a smooth shape for the brain, but in fact, the surface of the brain is folded. In a physical model that included folds, cavitation bubbles forced the walls of the fold apart, stretching the bottom of the fold (Kerwin et al. 2022). Post-traumatic tau protein pathology localizes to the bases of the folds in patients (Goldstein et al. 2012), and explaining this phenomenon is an important goal in modern TBI biomechanics (the shear strain theory can also explain it by describing it as a strain concentration effect (Braun et al. 2020; Ghajari et al. 2017)). In summary, physical models provide ample evidence that cavitation can occur in head impact-like scenarios. However, the clinical relevance of these results depends on the biofidelity of the models.

The cavitation pressure of degassed, filtered CSF is more than double that of degassed, filtered water (Bustamante & Cronin 2019). However, this difference may be of little consequence. The CSF in the brain is neither degassed nor filtered so nucleation sites probably set the threshold pressure for cavitation rather than the intrinsic properties of the fluid. The critical acceleration for cavitation in a gel greatly exceeds that of pure water (Kang et al. 2018), which helps explain why cavitation occurs on the surface of the brain but not inside it. Cavitation in gels also differs qualitatively from cavitation in water because cavitation bubbles in gels can transition from a spherical shape to a saucer-shaped void reminiscent of a crack in a brittle material (Kim et al. 2022). Barney et al. recently provided a thorough review of cavitation in gels (Barney et al. 2020). Physical models cannot reveal how much cavitation harms the brain, regardless of how well they reproduce it, because that requires a living system.

## The pathological consequences of cavitation

Proving that cavitation harms the brain is a two-part experimental challenge. The first part is quantifying and locating cavitation in a living test subject. The second is quantifying and locating subsequent trauma pathology in that subject. Head impact experiments have been conducted in primates in which the top of the skull was replaced with clear plastic to allow videography of the cranial vault during impact (Pudenz & Shelden 1946). Such a system could in theory be used to observe cavitation. However, these experiments were conducted at a time when TBI research in primates was more socially acceptable than it is now. The most popular large animal model of TBI in the modern era is the pig (Karlsson et al. 2019). The pig skull is very thick so creating a port in it through which the cranial vault could be observed would be difficult. Cavitation due to impact has been detected acoustically in an intact cadaveric pig head (Eckersley et al. 2021; Eckersley et al. 2020). This measurement has not thus far been reported in a living pig, but it is feasible in theory.

Measuring pressure fluctuations is easier than directly detecting cavitation. Cavitation limits how negative the pressure in a fluid can become so cavitation can be inferred when pressure reaches a negative limit. Striking fluid-filled human skulls created a pressure gradient that declined steadily along the line of action of the impact force until it reached a limiting value of approximately -1 atm (Lindgren 1964, 1966; Lindgren & Rinder 1966), which implies cavitation. The smaller the dimension of the skull along the line of action, the less likely the fluid pressure is to drop low enough to cause cavitation. It is, therefore, difficult to reproduce cavitation in convenient laboratory animals, which have much smaller skulls than humans (Lindgren & Rinder 1966). This problem can be solved by attaching a long column of fluid to a craniotomy and striking the top of the column. This approach created negative pressures and functional deficits in a rabbit model (Stålhammar 1975b, 1975a; Stålhammar & Olsson 1975). Nusholtz et al. detected negative pressures with a limiting value consistent with cavitation during head impacts in human cadavers and living primates. However, lesions did not develop in the primates in the regions where limiting negative pressures occurred (Nusholtz et al. 1984, 1987). Nevertheless, in subsequent work with physical and numerical models, Nusholtz concluded that cavitation could occur during a head impact if the loading was complicated by the presence of neck forces or rotational acceleration, and that the dura might serve to partially protect the brain from cavitation (Nusholtz, Benjamin Wylie, et al., 1995; Nusholtz, Wylie, et al. 1995a, b). In summary, animal models provide only indirect evidence of cavitation and associated pathology in TBI. Direct observation of cavitation and subsequent collocated pathology has so far been limited to in vitro models.

A range of in vitro models have been used to create cavitation injury in brain tissue. A split Hopkinson pressure bar passes a pressure wave through a sample to measure its mechanical properties at extremely high strain rates (Kolsky 1949). This device was used to load an organotypic brain slice embedded in gel. Cavitation bubbles formed near the tissue, tearing it and triggering neuronal degeneration (Sarntinoranont et al. 2012). The location of cavitation in this system can be controlled by introducing a seed bubble (Hong et al. 2016). When the bubble collapses, it tears any tissue within a few millimeters of it (Canchi et al. 2017). The flyer plate model involved directing a laser at a copper plate under a petri dish of cells, causing a fragment of copper to fly up into the bottom of the dish. The impact triggered a shock wave and cavitation event in the cell culture media (Cao et al. 2016). The imploding cavitation bubble instantly destroyed neural cancer cell line cells in a small region underneath it. The surviving cells reduced mitotic behavior and released S100B, a clinical biomarker of neurotrauma (Bjursten et al. 2024). Cavitation can also be induced in vitro using an electrical discharge (Sun et al. 2015). This model included two astrocyte monolayers, one above the electrodes and one below. A shock wave emanated from the discharge in both directions, but the cavitation bubbles only moved upwards. Therefore, the lower monolayer provided a convenient control proving that the shock waves do little damage unless they are accompanied by imploding bubbles. Again, cavitation destroyed astrocytes in a small region and injured adjacent cells, as evidenced by superoxide accumulation, cell death, and electrophysiological dysfunction (Kanagaraj et al. 2018). Poloxamer 188, an agent that helps reseal membranes, partially protected both astrocytes and epithelial cells in this model (Inyang et al. 2020), suggesting that membrane poration was an important mechanism of injury. The highly local nature of injuries created by cavitation is a recurring feature of in vitro models. In the electrical discharge model, the injury was confined to a domain within about 800 μm of the cavitation bubbles (Chen et al. 2019). Smaller bubbles are expected to have even more local effects (Adhikari et al. 2016). Molecular dynamics simulations predict that nanobubbles collapsing near cell membranes would create microjets that could perforate the plasma membrane (Choubey et al. 2011), damage membrane-bound channel proteins (Lau et al. 2016), or disrupt the tight junction proteins that knit together epithelial cells in the blood–brain barrier (Goliaei et al. 2015). In summary, in vitro studies agree that, when cavitation bubbles implode, they have a devastating effect on neural tissue within a small domain on the order of the size of the bubble. In light of these data, it is very likely that cavitation on the brain surface in vivo causes local injury.

## Discussion

To understand the place of cavitation in modern TBI biomechanics, we must first consider an important recent trend in the field: a shift in emphasis from severe TBI to mild TBI. Injury biomechanics began with the study of extremely high-energy events, specifically plane crashes (De Haven 1942). As the public and their governments came to expect car manufacturers to take more responsibility for occupant safety (Nader 1965), automotive crashes, which are also high-energy events, came to dominate the conversation. For most of the last century, concussion was considered inconsequential. However, in this century, evidence that mild TBIs can accelerate (Brett et al. 2022) or initiate (McKee et al. 2015) neurodegeneration has prompted alarm about mild TBI patients, who far outnumber moderate and severe TBI patients. The National Football League (NFL), the National Collegiate Athletic Association, and the Department of Defense in the US have invested heavily in understanding the biomechanics and pathology of mild TBI (Houston et al. 2020; Pellman 2003). Rapid progress has been made in how helmets are tested (Rowson & Duma 2011) and designed (Diekfuss et al. 2021) to minimize the risk of concussion. As attention to mild TBI has increased, attention to cavitation has decreased in the blunt impact TBI community (cavitation remains the subject of intense investigation in the blast TBI community as reviewed elsewhere (Marsh & Bentil 2021; Sundar & Ponnalagu 2021)). The attitudes of researchers to the cavitation and shear strain theories of traumatic brain injury can be inferred from the parameters they use to validate models and predict injury. Displacement and strain are the most important parameters according to the shear strain theory. Pressure is the most important parameter according to the cavitation theory. A recent attempt to predict concussions in reconstructed head impacts among NFL players relied exclusively on measures of strain and rotational kinematics (Sanchez et al. 2019). That study listed 12 preceding studies that used mechanical parameters to predict concussion. Six of those studies were published before 2007, and all six used both pressure (which indicates cavitation risk) and deviatoric strain as mechanical predictors. The other six studies were published after 2012, and none of those six considered pressure. Two types of cadaveric data exist for validating blunt impact FEMs of the human head: pressure data (Nahum et al. 1977; Trosseille et al. 1992) and displacement tracking data (Alshareef et al. 2018; Hardy et al. 2007). A recent review of human head FEMs for the study of TBI in contact sports criticized validation against pressure data on the grounds that ‘a model only “validated” against pressure is not sufficient for predicting large brain deformation generated from shearing of brain tissue’ (Ji et al. 2022). The possibility that a model only validated against displacement data is not sufficient for predicting pressures was not addressed.

Dismissing cavitation from the investigation of mild TBI may be a valid choice. Shear stress in the brain stem predicted concussion better than pressures in a computational model (Zhang et al. 2004), suggesting that cavitation is not the dominant mechanism. However, in most cases, protective equipment should not be designed exclusively to protect against concussion, nor should it necessarily be optimized for the most common impact scenario. Impact scenarios should be weighted based not only on frequency but also on outcome severity when optimizing performance. The case of football helmets is instructive in this regard. Football helmets were introduced in the 1970s, at a time when concussions were considered trivial injuries. Their original function was to prevent catastrophic, sometimes fatal head injuries, which were a significant problem at the time, and they largely succeeded in this regard (Boden et al. 2007). That function remains important. While most impacts on a football field are not life threatening, the ones that are deserve more attention than more common impacts that could cause concussion even though concussion is no longer considered a trivial injury. The current incomplete understanding of cavitation implies that preventing contusions, which are serious injuries that may be caused by negative pressure pulses and cavitation, should be considered as a distinct goal from preventing concussion, which are less dangerous injuries caused by deviatoric strains. These two goals could be addressed in standards and ranking schemes using different metrics, just as we use different metrics to assess the risk of neck injury and chest injury in car crash testing.

The practical consequences of this approach would be significant. For example, football helmets were traditionally made with stiff shells. Performance metrics based exclusively on concussion risk may guide designers to choose more compliant shells because this change reduces rotational acceleration. However, it is not clear that this change will have positive consequences for the risk of cavitation and contusion. A more compliant shell might mean more point loading of the skull during a rare event like getting kicked in the head. Several strands of evidence indicate that cavitation requires point loading and skull flexure. Analytical models with rigid ‘skulls’ did not produce pressure profiles consistent with cavitation but those with elastic ‘skulls’ did (see section on Analytical models of cavitation). Similarly, only those computational models that used point loading predicted cavitation (see section on Computational models of cavitation) and ultrasonic pressurization of physical models did not produce cavitation while impact did (see section on Physical modeling of cavitation). If point loading and skull flexure are indeed critical to cavitation, then making helmet shells more compliant might increase the likelihood of contusion in rare but severe impacts even as it decreases the risk of concussion in common but mild impacts. We cannot address this possibility without injury metrics for contusion based on its true mechanisms that are independent of the mechanisms and metrics used to understand concussion. More research into cavitation in blunt impact is needed to understand it as a mechanism and devise such metrics.

Overall, cavitation is an inconvenient but inescapable part of the biomechanics of blunt impact TBI. It is hard to detect, model, and quantify, and managing it may conflict with management of other well-established injury mechanisms. Nevertheless, the evidence that cavitation occurs in common head impact scenarios is compelling, as is the evidence that it harms brain tissue when it occurs, so ignoring it would be perilous.
